# Transcriptome comparison of dengue-susceptible and -resistant field derived strains of Colombian *Aedes aegypti* using RNA-sequencing

**DOI:** 10.1590/0074-02760200547

**Published:** 2021-05-28

**Authors:** Heather Coatsworth, Paola A Caicedo, Geoffrey Winsor, Fiona Brinkman, Clara B Ocampo, Carl Lowenberger

**Affiliations:** 1 Simon Fraser University, Department of Biological Sciences, C2D2 Research Group, Burnaby, BC, Canada; 2 Universidad Icesi, Natural Science Faculty, Centro Internacional de Entrenamiento e Investigaciones Médicas, Department of Biology, Vector Biology and Control, Cali, Colombia; 3 Simon Fraser University, Department of Molecular Biology and Biochemistry, Burnaby, BC, Canada

**Keywords:** dengue, Aedes aegypti, yellow fever mosquito, RNA sequencing, refractory mechanisms, innate immunity

## Abstract

**BACKGROUND:**

Forty percent of the world’s population live in areas where they are at risk from dengue fever, dengue hemorrhagic fever, and dengue shock syndrome. Dengue viruses are transmitted primarily by the mosquito *Aedes aegypti*. In Cali, Colombia, approximately 30% of field collected *Ae. aegypti* are naturally refractory to all four dengue serotypes.

**OBJECTIVES:**

Use RNA-sequencing to identify those genes that determine refractoriness in feral mosquitoes to dengue. This information can be used in gene editing strategies to reduce dengue transmission.

**METHODS:**

We employed a full factorial design, analyzing differential gene expression across time (24, 36 and 48 h post bloodmeal), feeding treatment (blood or blood + dengue-2) and strain (susceptible or refractory). Sequences were aligned to the reference *Ae. aegypti* genome for identification, assembled to visualize transcript structure, and analyzed for dynamic gene expression changes. A variety of clustering techniques was used to identify the differentially expressed genes.

**FINDINGS:**

We identified a subset of genes that likely assist dengue entry and replication in susceptible mosquitoes and contribute to vector competence.

**MAIN CONCLUSIONS:**

The differential expression of specific genes by refractory and susceptible mosquitoes could determine the phenotype, and may be used to in gene editing strategies to reduce dengue transmission.

Vector-borne pathogens are responsible for a significant proportion of the world’s most debilitating and devastating human diseases.^1^ In their role as vectors of protozoans, viruses, and nematodes, mosquitoes are the indirect cause of more than 2 million deaths annually.^2^ Of these, dengue is the most widespread arthropod-borne virus (arbovirus) disease, infecting up to 390 million people each year throughout tropical and subtropical regions.^1^ ^,^ ^2^ Dengue is transmitted primarily by *Aedes aegypti*, and to a lesser extent by *Aedes albopictus*. Changes in global travel, urbanization, and climate have facilitated the expansion of *Ae. aegypti* populations and consequently has allowed dengue to thrive. Half of the world’s population is at risk of contracting dengue, a statistic that could increase as the effects of climate change mount.^2^

Although insecticides, larvicides, and source reduction are used widely to reduce mosquito populations, none seem able to dampen dengue transmission significantly. This has resulted in an emphasis on mosquito bio-manipulation or genetic modification techniques to induce sterility, decrease lifespan, or reduce vector competence.^3^ These applications are based on understanding the molecular interactions between vector and virus, as well as basal vector genetics, and have shown great promise in developing new and effective vector control strategies.

Although *Ae. aegypti* is the principal vector of dengue viruses (DENVs), not all *Ae. aegypti* females transmit the virus. In Cali, Colombia, approximately 30% of field collected *Ae. aegypti* are refractory to all four dengue serotypes^4^ ^,^ ^5^ ^,^ ^6^ ^,^ ^7^ ^,^ ^8^ through one or more of the established barriers to flavivirus development; a midgut infection barrier (MIB) in which DENV is unable to replicate within midgut cells, or a midgut escape barrier (MEB) in which the virus cannot escape the midgut cells. Other barriers include a salivary gland infection barrier (SIB) in which the virus cannot enter the salivary glands, or a salivary gland escape barrier (SEB) in which DENV is unable to disseminate into the salivary gland lumen.^9^ These refractory mechanisms and overall innate immune responses to DENV have been studied principally in long established, specifically selected laboratory strains of *Ae. aegypti*.^10^ ^,^ ^11^ In Cali, Colombia we can collect mosquitoes in the field with one of three phenotypes; susceptible (Cali-S), refractory with a midgut infection barrier (Cali-MIB) and refractory with a midgut escape barrier (Cali-MEB). All three phenotypes can be collected within the same communities, and inside the same houses or oviposition sites within different neighborhoods. These have been raised in the laboratory and selected to increase the proportion of each phenotype, giving rise to the field derived strains.^8^

We have described significant differences in the expression of apoptosis related genes in the Cali-MIB and Cali-S strains.^6^ ^,^ ^7^ ^,^ ^8^ Knocking down apoptosis related genes altered the phenotype of *Ae. aegypti*, but could only explain ~30% of the refractory phenotype.^7^ ^,^ ^8^ We carried out a midgut transcriptome analysis, using RNA sequencing (RNA-seq) technology, to identify all transcripts in the midguts of Cali-S and Cali-MIB females at three different time points (24, 36 and 48 h post feeding) that are relevant to the period when the virus is entering, replicating in, and then exiting the midgut epithelial cells respectively. The aim of this study was to identify, in an unbiased manner, all differentially expressed genes that might contribute to the refractory or susceptible phenotypes.

Other studies have examined gene expression profiles after exposure to DENV in laboratory colonies of *Ae. aegypti* that are susceptible to DENV,^9^ ^,^ ^10^ ^,^ ^11^ and some have investigated semi-refractory laboratory strains.^9^ ^,^ ^10^ ^,^ ^12^ ^,^ ^13^ ^,^ ^14^ Our study is unique in that it analyzes mosquitoes that have evolved the refractory and susceptible phenotypes in the field with no human directed laboratory selection specifically for refractoriness or susceptibility to DENV.

## MATERIALS AND METHODS

*Ethics statement* - All female mosquitoes were exposed to dengue virus through an artificial membrane feeder. Adults in colonies were fed on hamsters at CIDEIM (Cali, Colombia) under protocols approved by the CIDEIM institutional review committee for research in animals (CIEIA).

*Mosquito rearing* - The collection, rearing and selection of the Cali-S and Cali-MIB strains of *Ae. aegypti* have been described.^8^ These strains were maintained under standard laboratory conditions: 28 ± 2ºC, 70% relative humidity, and a 12:12 h light-dark cycle. Adults were supplied with a 10% sugar solution *ad libitum*.

*Virus propagation and mosquito infections* - DENV-2 (New Guinea C strain) was propagated in *Ae. albopictus* (Skuse) C6/36HT cells. Infected cells were incubated for 14 days at 32ºC in L15 medium supplemented with 2% heat-inactivated fetal bovine serum (FBS), 1% penicillin/streptomycin, and 1% L-glutamine. Virus and cells were harvested and collected in a 15 mL conical centrifuge tube. The viral suspension was mixed 1:1 with defibrinated rabbit blood to create an infectious blood meal. Aliquots of the infected cell suspension, and the mixture of blood and virus were titred before and after the infection process as described previously.^8^ Titers in the cell suspensions ranged from 10^8^ to 10^8.5^ TCID_50_/mL in all oral challenges. Five to eight-day old adult female Cali-S and Cali-MIB mosquitoes were exposed for 2 h to the infectious blood meal via an artificial membrane feeder.^7^ All infections were carried out in Bio Safety Level 2+ facilities. After exposure to a blood meal with or without DENV-2, females that had fed to repletion were transferred to 300 mL containers, covered with mesh (~20 mosquitoes/container), and were given access to 10% sucrose solution *ad libitum.* Containers were maintained under the laboratory conditions described above.

*Mosquito dissections* - Midguts from F_15_ adult females were dissected from each strain (Cali-S and Cali-MIB) under each feeding treatment (blood meal or blood meal with DENV) and at each time point [24, 36 and 48 h post blood meal (PBM)] (Table I). Any remaining blood in the midgut was removed during dissection and the tissues were rinsed in 1X phosphate-buffered saline (PBS). In order to obtain enough RNA, midguts from three biological replicates were pooled for each of the 12 treatment groups. All dissections were performed in diethylpyrocarbonate (DEPC) sterile water on a cold table, and dissected tissues were immediately transferred to a microcentrifuge tube containing 200 µL of RNAlater^®^ Stabilization Solution (Ambion, Austin, Texas). All samples were subsequently transported from CIDEIM (Cali, Colombia) to Simon Fraser University (Burnaby, British Columbia), and stored at -20ºC.

**TABLE I:** Full factorial treatment design outlining all twelve experimental treatments (n = 36 for each treatment)

Hours PBM	24				36				48			
Treatment	Blood		Blood + DENV-2		Blood		Blood + DENV-2		Blood		Blood + DENV-2	
Strain Cali-	S	MIB	S	MIB	S	MIB	S	MIB	S	MIB	S	MIB

DENV: dengue viruses; MIB: midgut infection barrier; PBM: post blood meal.

*RNA extraction, library preparation, and RNA sequencing* - Total RNA was extracted from each pool of midguts and carcasses using Trizol (Sigma, Oakville, Ontario) as per the manufacture’s protocols. RNA concentrations were determined using a spectrophotometer (NanoDrop, ND-1000). Poly-A mRNA purification was performed with the Micro Poly A Purist Kit (Ambion, Austin, Texas) following the manufacturer’s protocols. From each mRNA sample, 100 ng was used to generate cDNAs using the Ultra RNA Library Prep Kit for Illumina (New England BioLabs, Ipswich, Massachusetts). All purification reactions were completed using AMPure XP Beads (Beckman Coulter, Brea, California). Fragment length analyses and overall library quality were completed on the final libraries at 2 nM using a Bioanalyzer (Agilent High Sensitivity Chip, Agilent Santa Clara, California). Libraries were sequenced at 100X depth as technical duplicates across multiple lanes using an Illumina miSeq platform at Fusion Genomics (Burnaby, BC).

**Fig. 1:**
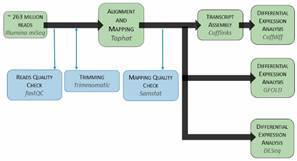
modified and adapted RNA rocket Galaxy portal bioinformatics workflow.

*Processing of raw sequencing reads* - A basic bioinformatics workflow was modified to accommodate newer programs and multiple analyses (Fig. 1). A complete list of all bioinformatic resources can be found in Supplementary data (Table I). The quality of the sequence data from each of the 12 treatments was checked using FastQC (v. 0.11.1), and a sequence trimmer, Trimmomatic (v. 0.30), was used on each of the 12 files to reduce overrepresented sequences, as well as to remove sequences less than 90 bp in length.

*Read alignment and mapping* - The *Ae. aegypti* genome and associated gene annotation files were obtained from VectorBase (http://www.vectorbase.org): AaegL3 Scaffolds was used as the genome, while AaegL3 Basefeatures was used for gene annotation. Tophat2 (https://tinyurl.com/yyxv8r3a) was used to align and map reads. Samstat (v.1.09) (https://tinyurl.com/yy9b7t5z) subsequently was used to check mapping quality.

*Differential expression tests* - Twenty-four separate differential expression (DE) tests were run to investigate the effects of time, viral presence and mosquito strain [Supplementary data (Table II)]. Because our MIB strain is only ~50% refractory, we assumed, based on Caicedo et al.,^8^ that 50% of the refractory mosquitoes were indeed phenotypically refractory. As such, strain comparisons were made between a pool of susceptible mosquitoes, and a pool of half refractory and half susceptible mosquitoes. Three different programs were used to analyze the RNA-seq data. Cuffdiff (v. 2.2.1) was used to test differential expression at both the gene and transcript level. Cuffdiff differential expression tests without replicates were run using the ‘blind’ method, while tests with replicates were run using the ‘pooled’ method. DESeq2 (v. 1.16.0), was also used to test for differential expression at the gene level. Tests without biological replicates were run under the ‘blind’ method, ‘fit-only’ sharing mode, and the ‘parametric’ fit-type, while tests with replicates were run using the ‘pooled’ method, the ‘maximum’ sharing mode, and the ‘parametric’ fit-type. Both programs generate a p-value from analyzing if the variance present in a group of samples is beyond what is expected from a simple Poisson model of the RNA sequencing data. Fold change values from Cuffdiff and DESeq2 are generated from Fragments Per Kilobase of transcript per Million mapped reads (FPKM) values, specifically, log_2_(FPKM_sample1_/FPKM_sample2_). A third differential expression program, GFOLD (v. 1.1.1), which is specifically designed for RNA-seq analyses without replicates, was used with default parameters. GFOLD reports a GFOLD value, which acts as a reliable log_2_ fold change value, calculated using Reads per Kilobase of transcript per Million mapped reads (RPKM) values. GFOLD values of zero show no differential expression. Sequences of differentially expressed genes were linked with annotations corresponding to gene names, GO (Gene Ontology) terms, and KEGG (Kyoto Encyclopedia of Genes and Genomes) terms in order to facilitate downstream over-representation analysis (annotations obtained using the Biomart tool at VectorBase).

*Analysis of DE data* - Two separate sub-analyses were completed on each DE output; one included only differentially expressed genes, and another that contained immune-related genes that were not differentially expressed statistically, but which the literature has indicted that even small changes in gene expression can have serious biological implications. To complete the immune specific analysis, an *Ae. aegypti* specific immune related list of genes was downloaded from ImmunoDB [Supplementary data (Table III)]. Functional classifications were assessed using a concatenated list of GO terms obtained through ImmunoDB. The second analysis was performed in a similar manner, excluding the ImmunoDB gene filtering step.

Clustering analyses and functional enrichment tests were completed on data obtained from all treatments, without incorporating a variance scaling factor, as data were found to be homoscedastic [Supplementary data (Fig. 1)]. To investigate how closely the expression profiles from each sample compared, a principal component analysis (PCA) plot using Euclidian distances was created using DESeq2 by log transforming the merged read count. *Ae. aegypti* GO terms were used to complete functional over-representation analyses via Ontologizer (v. 2.0). Two main types of clustering were performed through R (v. 3.1.1): hierarchical and partitioning (k-means) clustering. Dendrogram cutting was used to determine the optimal number of clusters for k-means clustering.

*Validation of differential gene expression using quantitative polymerase chain reaction (PCR)* - Droplet digital PCR (ddPCR) and quantitative real time PCR (qPCR) were used to validate expression values from RNA-seq. Validation tests were completed on cDNAs generated from three independently generated biological replicates from different generations of mosquitoes than those used to create the RNA-seq libraries. Thermocycling conditions for ddPCR were: 95ºC for 10s, 55ºC for 10 s, and 72ºC for 30 s in 20 µL reactions (containing 1 µL of cDNA) using QX200 ddPCR EvaGreen Supermix (Bio-Rad Laboratories, Hercules, California, USA) on an automated QX200 Droplet Digital PCR System (Bio-Rad Laboratories, Hercules, California, USA). QuantaSoft v1.7.4 (Bio-Rad Laboratories, Hercules, California, USA) was used to obtain an absolute expression quantification. qPCR was performed on a Light Cycler^®^ 96 system (Roche, Basel, Switzerland) using PerfeCTa SYBR^®^ (Quantabio, Massachusetts, USA). Thermocycling conditions for qPCR were: 95ºC for 10 s, 55ºC for 10 s, and 72ºC for 30 s in 10 µL reactions (containing 4 µL of 1:50 diluted cDNA). LightCycler^®^ 96 Application Software Version 1.1.1 (Roche, Basel, Switzerland) was used to obtain relative gene expression comparisons against a constitutively expressed housekeeping gene, 40 S ribosomal protein RPS17 (AAEL004175). Comparisons between ddPCR and log_2_ qPCR values and RNA-seq GFOLD001 values were made, noting the direction and magnitude of change. All RNA-seq GFOLD001 values were divided by the corresponding ddPCR or qPCR values in order to test the similarity between the two datasets.

## RESULTS

*Raw sequencing reads processing, alignment and mapping* - Each RNA-seq library generated between 14 and 28 million reads > 90 bp for each of the 12 treatments (Table I). Eighty three percent of all reads mapped to the genome (17% unmapped), and 63% of the mapped reads had an error rate of less than 0.001% [Supplementary data (Table IV).

**Fig. 2:**
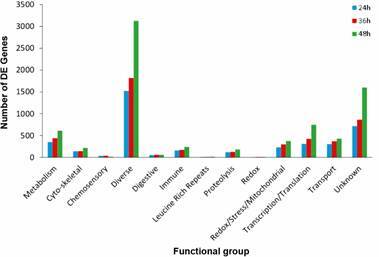
significantly differentially expressed genes between Cali-S and Cali-MIB strains at 24, 36 and 48 h after ingesting dengue virus serotype 2, arranged by broad functional groups denoted by ImmunoDB.

*Differential expression analysis* - All three programs identified the same differentially expressed genes. All time point comparisons under the Cali-S virus fed versus Cali-MIB virus fed test yielded similar functional group profiles (Fig. 2). Diverse and unknown functional groups represented the largest proportion of genes, followed by transcription/translation, transport, metabolism, redox/stress/mitochondrial and finally the immune group. The remaining groups each represented less than three percent of the total number of differentially expressed genes.

**Fig. 3:**
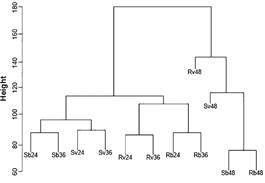
consensus hierarchical clustering result from DESeq2 (v. 1.16.0), Cuffdiff (v. 2.2.1) and GFOLD (v. 1.1.1) generated using R (v. 3.1.1). Clustering shows the phylogenetic relationship between all 12 treatment expression profiles (S: Cali-S, R: Cali-MIB, v: virus fed, b: blood fed, numbers represent time points). Euclidian distances were generated to compute Complete Linkage clustering.

*Statistical and systems analysis of differential expression data* - Hierarchical clustering produced a dendrogram (Fig. 3), with a clear separation between treatments analyzed at the 48-h time point (right branch) and all other treatments (left branch). The further splits within this right branch, were based on the viral treatment of the sample (either blood fed or blood and virus fed). The left branch however displayed an initial splitting of the Cali-S and Cali-MIB strains, with further branching into separate blood fed and virus fed treatments, and a final branching event into the 24 and 36 h time points.

The functional over-representation analysis again highlighted the 48 h time point as more diverse and dissimilar to the other time points. There was a large number of terms associated with cellular localization and transport across all comparisons. Comparisons at earlier time points (24 and 36 h) represented a generation of precursor metabolites, envelope proteins, and ion binding; while later time points (36 and 48 h), invoked intracellular signal transduction and small molecule metabolic processes [Supplementary data (Table V)].

Based on dendrogram cutting, the k-means analysis [Supplementary data (Fig. 2)] clustered all the gene count data into seven distinct clusters. The first cluster represented 98% of the genes (17,254), and as such, was tied to a wide variety of functional classes. Cluster 2 (44 genes) was primarily associated with ribosomal intracellular and translation functions, as well as RNA transport and degradation activity. Cluster 3 (one gene, AAEL013284) was specifically related to serine-type peptidase activity, as was cluster 7 (one gene, AAEL007818), and cluster 6 (10 genes). Similar to cluster 2, cluster 4 (162 genes) contained genes with many ribosomal functions, as well as functions associated with ATP binding and transport, metabolic pathways, and carbohydrate metabolism. Finally, cluster 5 (seven genes) was solely made up of genes representing metallopeptidases, GTP binding and GTPase activity.

*Choosing a candidate gene shortlist* - From these data, we further selected 15 differentially expressed genes (Table II) for further study based on three criteria: (i) highly differentially expressed, (ii) highly differentially expressed and immune related, and (iii) immune-related genes tagged in other published research papers (Table III). Genes were chosen from both the top significantly DE list, as well as the DE immune list. Only genes with documented or putative unique functions were chosen. Genes were classified as up-regulated when expression was higher in virus fed Cali-MIB versus virus fed Cali-S or blood fed Cali-MIB. Conversely, genes were labeled as down-regulated when expression was higher in virus fed Cali-S versus virus fed Cali-MIB or blood fed Cali-S. Up-regulated genes might be expressed to block viral cell entry and exit, stop replication or aid in immune viral clearance, while down-regulated genes may do the opposite, aiding in DENV entry, exit, and replication.

**TABLE II:** List of candidate genes

Gene ID	Gene name	Functional group	Up/Down	Function	Possible pathogen association	Reference
AAEL000693	TOR	Autophagy	Up	Inhibits autophagy, promoting cell growth	Inhibition should prevent viral replication	31
AAEL003712	C-type Lysozyme	Immunity	Up	Involved in innate immunity specifically in AMP expression	Lysozyme exerts a significant inhibitory effect on DENV	23
AAEL002360	Serine-type endopeptidase	Digestion	Up	Digestive enzymes which assists in breaking down meals	Proteolytic activity of serine-type endopeptidases limits virus infectivity	24
AAEL005444	Pyrokinin	Digestion and ionic balance	Up	Neuropeptide that regulates growth, metamorphosis, plays a role in anti-diuresis	Inhibits substrate absorption by anterior midgut	32 encodes a pyrokinin-related peptide (known as pyrokinin-1), PK1
AAEL002992	Sphingolipid delta 4 desaturase	Cell signaling	Up	Control cell proliferation, differentiation, and apoptosis		33
AAEL005641	C-type lectin	Binding	Up	Directly involved in cell galactose binding	Primary candidates for PRRs	34
AAEL001769	RM62B (dead box ATP-dependent RNA helicase)	Immunity	Up	Guides the silencing of target transcripts within small RNA pathway	Small RNA pathway (PIWI) may limit DENV replication	35
AAEL010195	Trypsin	Digestion	Up	Digestive enzyme which assists in breaking down meals	Higher trypsin expression results in higher DENV titers	25
AAEL013063	Autophagy related gene	Autophagy	Up	Participates in autophagy, an intracellular degradation system initiated upon stress	Autophagy plays a supportive role in DENV replication	27,36,37
AAEL002083	RM62C (dead box ATP-dependent RNA helicase)	Immunity	Up	Guides the silencing of target transcripts within small RNA pathway	Small RNA pathway may limit DENV replication	35
AAEL000028	CLIPB34	Immunity	Down	Serine protease involved in immune and developmental processes	Malaria parasites utilize CLIPs to sever surface proteins	38
AAEL014222	Low-density lipoprotein	Cell transport	Down	Mediates receptor endocytosis	A wide variety of viruses utilize LDLs to enter host cells	39
AAEL008083	40S ribosomal protein	Cell growth and proliferation	Down	Directly involved in protein translation	Putative receptor for the entry of DENV into host cells	26
AAEL009888	Bumetanide-sensitive Na-K-Cl cotransport	Transport	Down	Vital role in regulating ionic balance and cell volume		40
AAEL004978	RM62E (dead box ATP-dependent RNA helicase)	Immunity	Down	Guides the silencing of target transcripts within small RNA pathway	Small RNA pathway may limit DENV replication	35

Table includes VectorBase gene ID, gene name, functional group, general gene function, possible association with pathogens, and associated references. ‘Up’ refers to up-regulated in the Cali-MIB strain, while ‘Down’ refers to down-regulated in the Cali-MIB strain. AMP: anti-microbial peptide; DENV: dengue virus; LDL: low-density lipoprotein; PRR: pattern recognition receptor.

**TABLE III:** Shared genes between differentially expressed genes at 24 and 48 h. GFOLD differential expression values are displayed from this study, and compared with differential expression values. GFOLD value is the normalized GFOLD log_2_ fold change value, the first RPKM (reads per kilobase of transcript per million mapped reads values) represents the susceptible mosquitoes, while the second RPKM corresponds to the refractory mosquitoes. Panel A is a comparison after 24 h, while panel B is after 48 h

Vector base gene ID	GFOLD value	log2 fold change	FirstRPKM	SecondRPKM	Gene description
AAEL015458***	1.63429	2.09472	0.482571	4.29899	transferrin
AAEL008019**	1.15154	1.35835	8.45216	44.6763	hypothetical protein
AAEL006911*	1.13906	1.33454	2.89728	15.0603	microtubule-associated protein
AAEL005091**	0.962343	1.23939	2.49015	12.1359	conserved hypothetical protein
AAEL005561***	0.92409	1.06373	4.33529	18.661	plasma membrane calcium-transporting ATPase 3 (pmca3)
AAEL009762****	0.887696	1.40863	0.602798	3.33524	cytochrome P450
AAEL006138**^	0.83831	0.886424	19.4911	74.1502	hypothetical protein
AAEL010434**^	0.800671	0.867546	10.7857	40.5015	Vitellogenin-A1 Precursor (VG)(PVG1)
AAEL000940***	0.71424	0.768284	130.763	458.353	conserved hypothetical protein
AAEL008413**	0.709035	0.988036	1.21075	4.95468	serine/threonine protein kinase
AAEL001503*	0.659975	0.751801	8.16197	28.2886	sodium/hydrogen exchanger 3 (nhe3)
AAEL003609**	0.637787	0.917252	1.18354	4.61077	neurobeachin
AAEL003733**	0.588112	0.805074	0.806103	2.90214	hypothetical protein
AAEL007817**	0.571004	0.857399	0.659328	2.46414	hypothetical protein
AAEL008234***^	0.565555	1.03674	0.559988	2.38295	dishevelled
AAEL017241**	0.540569	0.922035	1.62698	6.37326	
AAEL006126**^	0.528522	0.597776	7.44993	23.2034	conserved hypothetical protein
AAEL006563**^	0.49487	0.670025	7.09483	23.2468	Vitellogenic carboxypeptidase Precursor (EC 3.4.16.-)
AAEL008216***	0.468322	0.548729	15.6068	46.9852	aconitase
AAEL003331***	0.389598	1.01092	0.214398	0.901404	hypothetical protein
AAEL008853***	0.363469	0.515272	7.3669	21.6768	choline/ethanolamine kinase
AAEL000191***	0.358468	0.492464	6.24993	18.0997	conserved hypothetical protein
AAEL006728***^	0.287245	0.577257	2.62092	8.06135	ubiquitin-conjugating enzyme E2 c
AAEL013074***^	0.281054	0.373851	20.7738	55.4011	adenylyl cyclase-associated protein
AAEL006785***	0.275308	0.314515	210.798	539.46	60S ribosomal protein L18a
AAEL009630**	0.267701	0.519107	1.76691	5.21699	high-affinity cgmp-specific 3,5-cyclic phosphodiesterase
AAEL005358****	0.266146	0.571726	0.924488	2.83316	conserved hypothetical protein
AAEL000087***	0.230243	1.8899	0.020329	0.178293	macroglobulin/complement
AAEL001972***	0.213241	0.571861	3.44608	10.5697	TATA box binding protein (TBP)-associated factor, putative
AAEL004699***	0.207845	0.258941	34.393	84.6913	conserved hypothetical protein
AAEL008329***	0.187807	0.239528	207.059	503.059	60S ribosomal protein L24
AAEL011326***^	0.170562	0.563302	0.751053	2.29116	conserved hypothetical protein
AAEL011756***	0.157017	0.213465	68.2496	162.847	aldehyde dehydrogenase
AAEL013614***^	0.138192	0.220292	8.36298	20.0499	clathrin heavy chain
AAEL005706***	0.133593	0.491582	0.733715	2.12793	triacylglycerol lipase
AAEL013694***	0.112597	0.149466	277.193	632.684	40S ribosomal protein SA
AAEL001898***	0.08628	0.243694	3.39235	8.26768	conserved hypothetical protein
AAEL006511***^	0.076473	0.117664	374.017	835.071	ubiquitin
AAEL001158***	0.035571	0.379164	1.78576	4.78794	fructose-1,6-bisphosphatase
AAEL001516***^	0.008267	0.172471	3.04556	7.06473	vesicle associated protein, putative
AAEL011900**	0.007043	0.398599	1.41796	3.85581	N-acetyllactosaminide beta-1,3-Nacetylglucosaminyltransferase, putative
AAEL000026***^	-0.02263	-0.02764	0.382792	0.775454	dynein light chain, putative
AAEL002813***	-0.02916	-0.15403	56.2173	103.968	coupling factor, putative
AAEL013252***	-0.08415	-0.34997	2.47179	3.98877	hypothetical protein
AAEL013407***^	-0.08858	-0.13579	64.4558	120.724	catalase
AAEL007293***^	-0.115	-0.28768	6.79592	11.4547	cAMP-dependent protein kinase catalytic subunit
AAEL011476***^	-0.13368	-0.6027	1.91153	2.58157	conserved hypothetical protein
AAEL009275***	-0.13769	-0.29124	12.5854	21.1615	protein phosphatase-1
AAEL009658***	-0.15448	-0.29219	6.7785	11.3903	alpha,alpha-trehalase
AAEL013979***	-0.21581	-0.6126	1.88957	2.5368	conserved hypothetical protein
AAEL015312****	-0.25461	-0.38054	23.8955	37.7669	cathepsin b
AAEL004181**	-0.26924	-0.45802	1.12191	1.68004	conserved hypothetical protein
AAEL002793***	-0.32904	-0.66303	1.2945	1.67913	conserved hypothetical protein
AAEL001432***	-0.34173	-0.41512	39.1863	60.4723	protein disulfide isomerase
AAEL012245****	-0.44171	-2.28002	0.326285	0.109783	conserved hypothetical protein
AAEL003067****	-0.45827	-1.09195	1.07278	1.02269	conserved hypothetical protein
AAEL002759***	-0.46062	-0.5438	17.766	25.0759	tropomyosin invertebrate
AAEL004958****	-0.46068	-3.56953	0.110887	0	conserved hypothetical protein
AAEL012349****	-0.46068	-3.56953	0.154968	0	lipase 1 precursor
AAEL013566****	-0.48876	-1.91745	0.978856	0.494022	C-Type lectin (CTL) - galactose binding
AAEL015004***	-0.51611	-0.79771	3.27493	3.87027	hypothetical protein
AAEL004027***	-0.59755	-0.70559	26.8693	33.8975	glucose dehydrogenase
AAEL014190****	-0.59899	-2.52514	0.189295	0.048122	elongase, putative
AAEL009244***	-0.61831	-0.6692	195.675	253.206	serine-type enodpeptidase
AAEL013853****	-0.92413	-1.80399	0.947571	0.535306	C-Type Lectin (CTL) - galactose binding
AAEL013648****	-0.93159	-2.59552	0.293348	0.08286	conserved hypothetical protein
AAEL001295****	-1.03599	-1.58042	1.66246	1.1286	conserved hypothetical protein
AAEL002652***	-1.14538	-2.98457	0.129852	0.023579	hypothetical protein
AAEL007942***^	-1.25268	-1.58534	7.32334	4.99762	fibrinogen and fibronectin
AAEL017211**	-1.36322	-1.89536	16.8411	9.17417	cecropin anti-microbial peptide
AAEL001287**	-1.5998	-3.38312	0.620966	0.084568	conserved hypothetical protein
AAEL002796**	-1.78703	-2.66264	0.730636	0.224168	l-asparaginase i
AAEL008046**	-2.28638	-2.73068	4.00053	1.22289	rh antigen
AAEL003290**	-3.47153	-6.25985	1.0477	0	cell wall protein DAN4 precursor, putative
AAEL017110**	-5.13546	-7.88489	7.924	0	
AAEL009888**	-5.69755	-7.44101	1.44731	0.008596	bumetanide-sensitive Na-K-Cl cotransport protein, putative

Vector base gene ID	GFOLD value	log2 fold change	FirstRPKM	SecondRPKM	Gene description
AAEL008392***^	0.812048	1.3925	0.932716	2.58007	conserved hypothetical protein
AAEL006291***	0.57612	0.754547	4.46132	7.84022	cullin
AAEL010798***^	0.303677	0.440852	14.8129	20.9368	ubiquitin-conjugating enzyme E2 g
AAEL000604***^	0.281466	0.478478	4.90627	7.11891	hypothetical protein
AAEL014190****	-0.07837	-2.18723	0.160454	0.020175	elongase
AAEL001295****	-0.10263	-0.4388	4.09937	3.14096	conserved hypothetical protein
AAEL004809****	-0.12691	-0.78669	2.839	1.68835	conserved hypothetical protein
AAEL002908****	-0.27134	-1.89772	1.03566	0.250423	hypothetical protein
AAEL002818***	-0.31054	-0.62888	3.96005	2.6588	splicing factor u2af large subunit
AAEL014035***^	-0.43611	-0.7278	2.89018	1.81228	suppressor of actin (sac)
AAEL002889****	-0.51237	-1.01956	4.71438	2.3963	hypothetical protein
AAEL007075***^	-0.75562	-1.15049	3.75319	1.74852	conserved hypothetical protein
AAEL001737****	-0.97528	-2.78669	0.35227	0.036911	conserved hypothetical protein
AAEL008546****	-1.02804	-3.97572	1.07999	0	conserved hypothetical protein
AAEL012326***	-1.08918	-1.18633	234.609	107.293	calmodulin
AAEL002696****	-1.18611	-1.73636	1.56737	0.480957	hypothetical protein
AAEL002023***^	-1.53962	-1.7826	12.42	3.74596	imaginal disc growth factor
AAEL011851***^	-2.09883	-3.61532	1.81474	0.125029	conserved hypothetical protein

^: previously detected as differentially expressed (see below for more information), but our results show changes in the opposite direction; *: previously detected as differentially expressed in mosquitoes of the MOYO-S or MOYO-R strains infected with DENv2 Jam1409 18 h post infection;^10^ **: previously detected as differentially expressed in Chetumal (CTM) mosquito midguts 1dpi with DENv2 Jam1409 or blood;^11^ ***: previously detected as differentially expressed in mosquitoes of the MOYO-S or MOYO-D strains infected with DENv2 Jam1409 24 h post infection;^14^ ****: previously detected as differentially expressed in mosquitoes of the Rockefeller strain infected with DENv2 New Guinea C 48 h post infection.^12^

^: previously detected as differentially expressed (see below for more information), but our results show changes in the opposite direction; ***: previously detected as differentially expressed in mosquitoes of the MOYO-S or MOYO-D strains infected with DENv2 Jam1409 48 h post infection;^14^ ****: previously detected as differentially expressed in mosquitoes of the Rockefeller strain infected with DENv2 New Guinea C 48 h post infection.^12^

*Differential expression validation* - Four candidate genes (autophagy related target of rapamycin, TOR, AAEL000693, a 40S ribosomal gene, AAEL013694, a low-density lipoprotein receptor gene, AAEL014222, and a bumetanide-sensitive Na-K-Cl co-transport, AAEL009888), and five non-candidate genes (60 S ribosomal protein L15, AAEL012736, 60 S ribosomal protein L35a, AAEL000823, Eukaryotic translation initiation factor 3 subunit G, AAEL012661, an uncharacterized gene, AAEL002930, and 4-nitro, AAEL007097) were chosen as representatives for differential expression validation. ddPCR was used to validate candidate genes, as their expression levels were lower and this technique is more sensitive than qPCR. Non-candidate gene validation was completed using qPCR [Supplementary data (Fig. 3)], as the overall expression levels of these genes was high enough for reliable detection. Any genes that differed in direction of level of differential expression between RNAseq and ddPCR analyses (AAEL013694, AAEL014222 and AAEL000693) were removed from the candidate gene list.

## DISCUSSION

*Differential expression analysis* - All three methods (Cuffdiff, DESeq2 and GFOLD) identified the same top differentially expressed genes. Cuffdiff and DESeq2 are both extremely conservative in their list of differentially expressed genes compared to GFOLD. It is not uncommon for immunologically relevant genes to rank below others in the list of the greatest differentials in gene expression. This trend is further highlighted in immune genes, where even slight changes in expression may have large downstream effects. As such, the ordering of gene expression differences (from highest to lowest) may have resulted in ranking biologically relevant genes lower in importance based solely on expression level differences. To overcome this quandary, two lists of candidate genes were generated: one based on the top DE genes (shared amongst all three programs), and one based on the most expressed immune related genes (as identified by ImmunoDB, still shared amongst all three programs).

There were some general trends within the dataset. Both Cali-S and Cali-MIB mosquitoes infected with DENV-2 had increased expression of digestive genes such as trypsins, serine endopeptidases and metalloproteinases compared with their counterparts fed solely on blood. These digestive enzymes are likely important early regulators of infection;^15^ an increase in these digestive enzymes could assist in dampening the ability of DENV to enter and replicate in cells, as the level of viral degradation within the midgut could be higher.

In Cali-MIB females exposed to DENV, we observed higher levels of metalloproteinases (MMPs), as well as increases in the expression of a Niemann-pick type C2 gene. Niemann-pick-C2 is a cholesterol transporter, and has been identified in various studies as a viral agonist that may enhance, or be required for, the entry of DENV-2 into cells.^16^ However, our results in refractory mosquitoes seem to suggest the opposite. It is possible, that in response to other mechanisms expressed to decrease viral titres in the midgut of refractory mosquitoes, that DENV upregulates Niemann-pick C2 expression to remain viable.

In Cali-S females, we see higher expression levels of several odorant binding proteins (OBPs) (AAEL006176-OBP27, AAEL002606-OBP35, AAEL012377-OBP55, AAEL009449-OBP39, AAEL010666-OBP42, AAEL013018-OBP56) as well as an anti-apoptosis gene (AAEL009074-Inhibitor of Apoptosis, AeIAP1). The role of OBPs in the midgut is unclear, although it has been proposed that they act as signalling mechanisms for odorant binding proteins in the salivary glands,^17^ inducing the mosquito to bite repeatedly and enhancing virus transmission. *AeIAP1*, on the other hand, is involved in inhibiting apoptosis, and has been characterized as being pro-viral, preventing infected cells from undergoing apoptosis and eliminating the virus before it replicates. Unfortunately, knock-downs of *AeIAP1* were lethal to the mosquitoes, and cannot be the sole mechanism driving refractoriness in the Cali-MIB strain.

There were notable temporal differences in the expression of genes within each treatment and strain. Most digestive function ontology terms correlate directly with mosquito blood meal processing. We observed trypsins and sodium and potassium co-transporters at 24 h post blood meal, serine endopeptidases, carboxypeptidases and lipases at 36 h PBM, and heme peroxidases, cytochrome p450s and sucrose transporters at 48 h PBM. Insects rapidly produce digestive enzymes upon feeding, and these decrease in production as absorption occurs within the midgut.^18^ Specifically, late trypsin is activated 12-48 h post blood meal, during which lipid digestion occurs via phospholipases and phosphatases, which hydrolyze ester bonds, solubilizing cell membranes for the passage of lipids into the hemolymph.^18^ This may result in the spike of expression in these enzymes at earlier time points, and lower expressions at later time points. The digestion of the blood meal produces toxic heme as a by-product, and this toxic heme has specific binding sites on the peritrophic matrix where it is bound and excreted after blood digestion has occurred. As a result, heme cannot interact with and damage the midgut epithelial cells. Mosquitoes also use p450-like enzymes such as CYP6 and CYP9 to assist in heme detoxification.^18^ This is likely why higher levels of heme peroxidases and cytochrome p450s start to appear around 48 h PBM. Sucrose transporters may have higher expression levels at 48h as the mosquito is likely in the process of digesting, and subsequently transporting, these carbohydrates.

Eighty five of the genes identified in this RNA sequencing study have been implicated in previous refractory mosquito expression studies,^10^ ^,^ ^12^ ^,^ ^13^ ^,^ ^19^ including a microarray study on Cali-MIB and Cali-S at one time point only; 30 h after blood feeding.^20^ These correlations centered on digestive genes such as trypsins and serine-endopeptidases, as well as signalling and cell entry genes such as lectins and lipoproteins. A smaller subset of immune related genes was also common, including a variety of anti-microbial peptides, CLIP (N-terminal to the chymotrypsin serine protease domain, named due to its likeness in shape to a paperclip) domains, apoptotic genes, and small RNA pathway molecules. Although many genes were common between these datasets, some genes had differences in their direction of differential expression. These differences were mostly evident between our refractory strain (Cali-MIB) and the MOYO-D strain, and primarily encompassed cell signalling, processing and transport genes such as ubiquitin, dynein, adenylyl cyclase, clathrin, dishevelled, and multiple vitellogenin precursors.

A large proportion of differentially expressed genes are MMPs, which have a strong association with immunity. MMPs play important roles in pathogen infection, acting as both agonists and antagonists. In humans, increased MMP activity is associated with increased pathogenicity, as MMPs assist in breaking down the basal lamina of tissues, allowing for subsequent viral entry and replication, often resulting in increased vascular leakage.^21^ In mosquitoes, MMPS have been implicated in extracellular matrix remodelling, potentially allowing virions to pass through an altered basal lamina.^22^ MMPs also have been annotated as anti-viral, acting as apoptotic effectors in the important anti-viral JAK-STAT pathway.^15^ In theory, decreasing these pervasive midgut specific MMPs would help insects create a tissue specific barrier to migrating pathogens. A down regulation of MMPs in *Ae. aegypti* was correlated with decreased viral titre, or elimination of DENV.^9^ ^,^ ^13^ ^,^ ^19^

We have reported trends observed within the dataset. Some genes in the *Ae. aegypti* genome have not been annotated, and their expression will appear in the dataset as ‘conserved hypothetical proteins’ or ‘hypothetical proteins’, and as such, the role of these genes was not examined.

*Statistical and systems analysis of differential expression data* - The outputs from the hierarchical clustering and PCA analysis were, as expected, similar, as both involve distance matrix measures to scale and visualize data sets. In both analyses the 48-h time point appears much more isolated than the 24- and 36-h time points, likely because the mosquito has finished or is near the end point of blood digestion, and thus different regulatory genes are at play. In Cali-S mosquitoes, by 48 h, the virus will have migrated into and replicated within other mosquito tissues, and as such, different genes may be expressed. Conversely, DENV does not enter the hemocoel of Cali-MIB mosquitoes and therefore it is likely that these expression differences may be related to the process of viral elimination. Furthermore, the lack of significant differences in Cali-S and Cali-MIB females fed solely on blood at 48 h PBM suggests that the major differences between these strains are directly related to their response towards DENV.

The partitioning (k-means) analysis allowed us to view the functional clustering of the differentially expressed genes, while the Ontologizer completed a functional over-representation analysis. Both outputs yielded similar trends. We observed many genes with a wide variety of functions, with many genes at 24 h and 36 h PBM associated with blood meal processing, suggesting differences in blood meal digestion between the two mosquito strains. Many ribosomal, RNA transport and degradation terms clustered together, which could suggest dengue is utilizing host mechanisms to help in its own replication. Since the virus itself utilizes the host endoplasmic reticulum for transport and assembly, these changes in expression could reflect efficient viral infection and propagation.

*Candidate gene analysis* - Most of these candidate genes (5/15) were immune related; a c-type lysozyme (AAEL003712), and three dead box ATP dependant RNA helicases (AAEL001769, AAEL002083, and AAEL 004978) may limit DENV replication as part of the mosquito’s innate immune response,^23^ while CLIBB34 (AAEL000028) may be manipulated by the virus to interfere with cell surface proteins. Multiple digestive genes (a serine-type endopeptidase, AAEL002360, a pyrokinin, AAEL005444, and a trypsin, AAEL010195), also in the list, have been proposed to limit viral infectivity due to their high proteolytic activity and role in absorption,^24^ although some studies have shown the opposite to be true.^25^ Candidate genes related to cell signaling, growth, binding and transport (a sphingolipid delta 4 desaturase, AAEL002992, a 40 S ribosomal protein, AAEL008083, a c-type lectin, AAEL005641, and a low-density lipoprotein, AAEL014222, respectively) may play roles in assisting or inhibiting viral cell entry.^26^ Lastly, two autophagy candidate genes, an autophagy related target of rapamycin, AAEL000693, and an autophagy related gene, AAEL013063, both up-regulated in Cali-MIB mosquitoes may play a role in viral replication.^27^ A summary of all our candidate genes, their function as well as possible DENV association can be found in Table II. We have focused on four of these genes (validated using ddPCR) in more detail below.

Although NaK (a bumetanide-sensitive Na-K-Cl co-transport, AAEL009888) has not been reported previously as important in the mosquito-dengue literature, it plays a vital role in regulating ionic balance and cell volume. NaK may be localized in the apical membrane of midgut epithelial cells in *Ae. aegypti*, as was demonstrated by an ortholog of AAEL009888 in *Manduca sexta*.^28^ Furthermore, sodium transporters are needed to maintain intracellular pH, and changes in the expression of these transporters could result in changes to cell homeostasis. NaK could be necessary for the maintenance of intracellular homeostasis, and this could be why we see a higher expression of the NaK transcript in susceptible infected mosquitoes.

Conversely, we observed a higher transcript expression of CTL, a c-type lysozyme (AAEL003712) in refractory mosquitoes. Lysozymes have historically been implicated as anti-bacterial agents. When lysozyme-c was silenced, mosquitoes had a higher titre of dengue virus, suggesting that lysozymes may exert an inhibitory effect on the virus itself.^23^

We found higher expression of autophagy related genes, which are normally associated with organelle recycling and destruction, but recently have been implicated in reducing viral titres.^29^ The opposite seems to be true for DENV infections, where autophagy related genes (APGs) augment viral infection and replication.^27^ Silencing *Aedronc*, an initiator caspase, decreased autophagy and DENV titres in *Ae. aegypti*, suggesting an apoptotic basis of autophagy control.^29^ DENV may induce autophagy and subsequent autophagosome formation, using virus induced double membrane vesicles as replication sites,^27^ although mechanisms using lipid metabolism, lipid droplets, virion maturation and dsRNA localization also have been proposed.^15^ This pro-viral effect is consistent with reports describing significant increases in APG expression in susceptible mosquitoes exposed to DENV.^7^ ^,^ ^9^ ^,^ ^11^ ^,^ ^13^ There is a trend in DENV-refractory mosquitoes to have increased expression of Inhibitor of Apoptosis (IAP), Buffy, and anti-apoptotic genes,^9^ ^,^ ^13^ suggesting that the autophagy pathway may contribute to the DENV refractory phenotype.

A limitation of this study is that the experiments were not fully replicated; material from multiple replicates were pooled for the RNA sequencing, and therefore we analyzed the data with multiple programs and approaches for replicated and non-replicates experiments. All approaches identified the same genes. We identified specific genes that were over- or under-expressed in Cali-MIB or Cali-S mosquitoes after exposure to dengue virus. The results on the differential expression of specific genes identified using the RNAseq approach were confirmed using cDNAs generated from the same RNAs used to make the libraries, but also were confirmed using cDNAs generated from independently selected MIB and S lines of mosquitoes.

It is evident that there are proximate differences in DENV processing by Cali-S and Cali-MIB females, although the rationale is unclear because most studies suggest very little or no significant impact of DENV on overall *Ae. aegypti* fitness.^30^ Whether these responses are restricted to DENV, to other flaviviruses such as Zika, and yellow fever or to other arboviruses such as Chikungunya, will help us understand the extent of differential gene expression as a general antiviral response in *Ae. aegypti*. Future studies will use RNAi based gene knockdown studies to examine the phenotypic function of candidate genes identified in this study. We also will use DNA-based genetic analyses to separate inherent genetic differences between the strains from their differential responses to DENV.

*Data availability* - All relevant data are within the paper and its Supplementary data. The raw sequencing data as well as processed differential expression data is available to the public through NCBI’s Gene Expression Omnibus (GEO) database (GSE90974).
