# Supplementary material for: Transcriptome comparison of dengue-susceptible and -resistant field derived strains of Colombian Aedes aegypti using RNA-sequencing
Source: Mem Inst Oswaldo Cruz. 2021 May 28;116:e200547. doi: 10.1590/0074-02760200547 (PMC8186470; doi:10.1590/0074-02760200547)
Supplement: Supplementary file 1 [file 1678-8060-mioc-116-e200547-s.pdf]

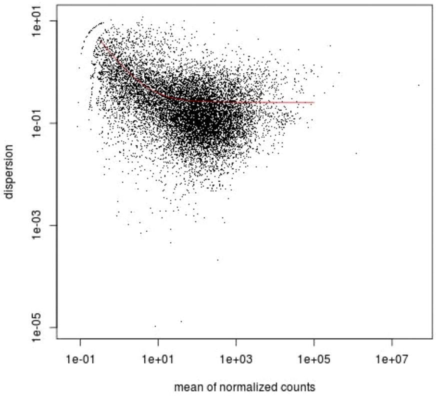

Fig. 1: RNA-seq heteroskedastic mean and variation distribution. The homoscedastic data test results are shown displaying the correlation between variance within data and mean. Each black point represents a gene expression count, and the red line represents the mean fitted value.

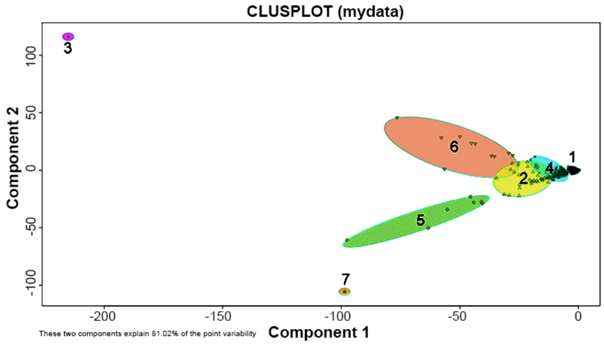

Fig. 2: K-means (partitioning) clustering on all gene expression data using R (v. 3.1.1). Cluster 1 (purple) represented a wide variety of functional classes, Clusters 2 (yellow) and 4 (teal) were associated with ribosomal intracellular and translation functions, Clusters 3 (fuchsia), 6 (red) and 7 (orange) all relate to serine-type peptidase activity, and Cluster 5 (green) was made up of mostly metallopeptidases.

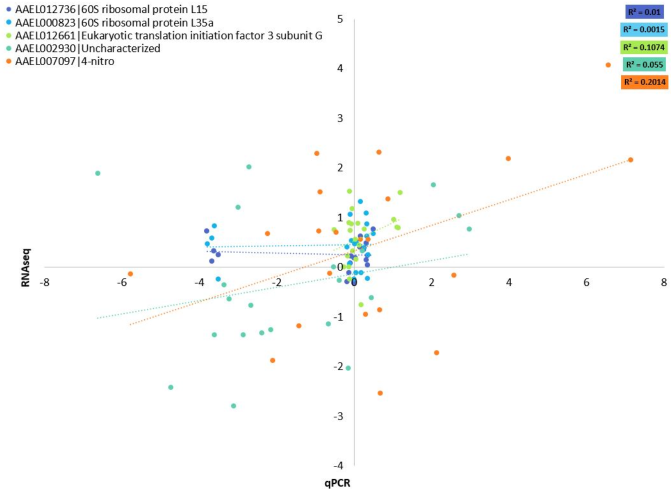

Fig. 3: comparison between quantitative polymerase chain reaction (qPCR) validation and RNAseq data for non-candidate genes. A scatter plot of qPCR (y-axis) and GFOLD RNAseq values (x-axis) displays the expression values for non-candidate genes. An R^2 value and line of best fit of these relationships is shown on the figure displaying the overall concordance between the two datasets for each gene.

CC BY

TABLE I

A complete list of all informatics programs and databases used in the manuscript. Lists all versions and citations.

Informatics program	Reference
Galaxy portal	Blankenberg D, Von Kuster G, Coraor N, Ananda G, Lazarus R, Mangan M, et al. Galaxy, a web-based genome analysis tool for experimentalists. Curr Protoc Mol Biol. 2010; 29(6): 997-1003.
FastQC (v.0.11.1)	Andrews S. FastQC: a quality control tool for high throughput sequence data. 2010; Available from: http://www.bioinformatics.babraham.ac.uk/projects/fastqc
Trimmomatic (v.0.30)	Bolger A, Lohse M, Usadel B. Trimmomatic: a flexible trimmer for Illumina sequence data. Bioinformatics. 2014; 30(15): 2114-20.
VectorBase	Megy K, Emrich SJ, Lawson D, Campbell D, Dialynas E, Hughes DST, et al. VectorBase: improvements to a bioinformatics resource for invertebrate vector genomics. Nucleic Acids Res. 2012; 40(D1): 729-34.
Tophat2	Kim D, Pertea G, Trapnell C, Pimentel H, Kelley R, Salzberg SL. TopHat2: accurate alignment of transcriptomes in the presence of insertions, deletions and gene fusions. Genome Biol. 2013; 14(4): R36.
Samstat (v.1.09)	Lassmann T, Hayashizaki Y, Daub C. SAMStat: monitoring biases in next generation sequencing data. Bioinformatics. 2011; 27(1): 130-1.
Cuffdiff (v.2.2.1)	Trapnell C, Williams BA, Pertea G, Mortazavi A, Kwan G, Van Baren MJ, et al. Transcript assembly and quantification by RNA-Seq reveals unannotated transcripts and isoform switching during cell differentiation. Nat Biotechnol. 2010; 28(5): 511-5.
DESeq2 (v.1.16.0)	Anders S, Huber W. Differential expression analysis for sequence count data. Genome Biol. 2010; 11(10): R106.
GFOLD (v.1.1.1)	Feng J, Meyer C, Wang Q, Liu J, Liu X, Zhang Y. GFOLD: a generalized fold change for ranking differentially expressed genes from RNA-seq data. Bioinformatics. 2012; 28(21): 2782-8.
GO (gene ontology) terms	Ashburne M, Ball C, Blake J, Botstein D, Butler H, Cherry J, et al. Gene ontology: tool for the unification of biology. Nat Genet. 2000; 25(1): 25-9.
KEGG (Kyoto Encyclopedia of Genes and Genomes) terms	Kanehisa M, Goto S. KEGG: Kyoto encyclopedia of genes and genomes. Nucleic Acids Res. 2000; 28(1): 27-30.
ImmunoDB	Waterhouse RM, Kriventseva EV, Meister S, Xi Z, Alvarez KS, Bartholomay LC, et al. Evolutionary dynamics of immune-related genes and pathways in disease-vector mosquitoes. Science. 2007; 316(5832): 1738-43.
Ontologizer (v.2.0)	Bauer S, Grossmann S, Vingron M, Robinson P. Ontologizer 2.0 - a multifunctional tool for GO term enrichment analysis and data exploration. Bioinformatics. 2008; 24(14): 1650-1.
R (v.3.1.1.)	Team RDC. R: a language and environment for statistical computing. 2011. R Foundation for Statistical Computing, Vienna.

TABLE II

An overview of all 24 differential expression analyses completed. A list of what samples were compared under each different analysis type, separating out the effect of time, virus and strain.

Type of differential analysis	Samples to compare		Biological replicates
Time series (within condition and strain) *isolating the effect of time	S1 (Sb24)	S2 (Sb36)	No
	S2 (Sb36)	S3 (Sb48)	No
	S1(Sb24)	S3 (Sb48)	No
	S4 (Sv24)	S5 (Sv36)	No
	S5 (Sv36)	S6 (Sv48)	No
	S4 (Sv24)	S6 (Sv48)	No
	R7 (Rb24)	R8 (Rb36)	No
	R8 (Rb36)	R9 (Rb48)	No
	R7 (Rb24)	R9 (Rb48)	No
	R10 (Rv24)	R11 (Rv36)	No
	R11 (Rv36)	R12 (Rv48)	No
	R10 (Rv24)	R12 (Rv48)	No
Time points (between conditions within strain) *isolating the effect of virus	S1 (Sb24)	S4 (Sv24)	No
	S2 (Sb36)	S5 (Sv36)	No
	S3 (Sb48)	S6 (Sv48)	No
	R7 (Rb24)	R10 (Rv24)	No
	R8 (Rb36)	R11 (Rv36)	No
	R9 (Rb48)	R12 (Rv48)	No
Time points (within condition and across strain) * isolating the effect of strain	S4 (Sv24)	R10 (Rv24)	No
	S5 (Sv36)	R11 (Rv36)	No
	S6 (Sv48)	R12 (Rv48)	No
Between conditions (across all times and within a strain) * isolating the effect of virus	S1 (Sb24),S2 (Sb36),S3 (Sb48)	S4 (Sv24),S5 (Sv36),S6 (Sv48)	Yes
	R7 (Rb24), R8 (Rb36), R9 (Rb48)	R10 (Rv24), R11 (Rv36), R12 (Rv48)	Yes
Between strains (across all times and within a condition) * isolating the effect of strain	S4 (Sv24), S5 (Sv36), S6 (Sv48)	R10 (Rv24), R11 (Rv36), R12 (Rv48)	Yes

TABLE III
The list of genes classified as immune related, as mined from ImmunoDB. Only genes with a confirmed and high confidence status (A and B) were kept for classification.

Gene ID	Name	Subfamily	Family
AAEL013815	APG10	APHAG	Autophagy genes
AAEL009089	APG12	APHAG	Autophagy genes
AAEL013063	APG18A	APHAG	Autophagy genes
AAEL013995	APG18B	APHAG	Autophagy genes
AAEL003799	APG2	APHAG	Autophagy genes
AAEL000955	APG3	APHAG	Autophagy genes
AAEL010516	APG4A	APHAG	Autophagy genes
AAEL007228	APG4B	APHAG	Autophagy genes
AAEL002286	APG5	APHAG	Autophagy genes
AAEL010427	APG6	APHAG	Autophagy genes
AAEL010641	APG7A	APHAG	Autophagy genes
AAEL012306	APG7B	APHAG	Autophagy genes
AAEL007162	APG8	APHAG	Autophagy genes
AAEL009105	APG9	APHAG	Autophagy genes
AAEL001521	BUFFY	APHAG	Autophagy genes
AAEL001515	DEBCL	APHAG	Autophagy genes
AAEL000693	TOR	APHAG	Autophagy genes
AAEL015246	AGO1A	Argonaute	Small regulatory RNA path
AAEL012410	AGO1B	Argonaute	Small regulatory RNA path
AAEL007823	AGO3	Argonaute	Small regulatory RNA path
AAEL000872	ARK	ARK	Caspase activators
AAEL010693	ARM1	Armitage	Small regulatory RNA path
AAEL010696	ARM2	Armitage	Small regulatory RNA path
AAEL003389	ATT	Attacin	AMPs
AAEL000709	CACT	CACT	TOLLPATHS
AAEL014738	CASPAR1	CASPAR	IMDPATHs
AAEL003579	CASPAR2	CASPAR	IMDPATHs
AAEL014148	CASPL1	CASPL	Caspases
AAEL011562	CASPL2	CASPL	Caspases
AAEL005963	CASPS15	CASPS	Caspases
AAEL005956	CASPS16	CASPS	Caspases
AAEL003439	CASPS18	CASPS	Caspases
AAEL003444	CASPS19	CASPS	Caspases
AAEL014658	CASPS20	CASPS	Caspases
AAEL014348	CASPS8	CASPS	Caspases
AAEL013407	CAT1B	CAT	Catalases
AAEL000627	CECA	Cecropin	AMPs
AAEL004223	CECB	Cecropin	AMPs
AAEL000598	CECD	Cecropin	AMPs
AAEL000611	CECE	Cecropin	AMPs
AAEL000625	CECF	Cecropin	AMPs
AAEL015515	CECG	Cecropin	AMPs
AAEL000775	CECI	Cecropin	AMPs
AAEL000777	CECJ	Cecropin	AMPs
AAEL000621	CECN	Cecropin	AMPs
AAEL002601	CLIPA1	CLIPA	CLIPs
AAEL001675	CLIPA10	CLIPA	CLIPs
AAEL002126	CLIPA15	CLIPA	CLIPs
AAEL008404	CLIPA16	CLIPA	CLIPs
AAEL007006	CLIPA17	CLIPA	CLIPs
AAEL005718	CLIPA3	CLIPA	CLIPs

AAEL000074	CLIPB1	CLIPB	CLIPs
AAEL003243	CLIPB13A	CLIPB	CLIPs
AAEL003253	CLIPB13B	CLIPB	CLIPs
AAEL014349	CLIPB15	CLIPB	CLIPs
AAEL005648	CLIPB16	CLIPB	CLIPs
AAEL000059	CLIPB19	CLIPB	CLIPs
AAEL001084	CLIPB21	CLIPB	CLIPs
AAEL008668	CLIPB22	CLIPB	CLIPs
AAEL012785	CLIPB23	CLIPB	CLIPs
AAEL014140	CLIPB24	CLIPB	CLIPs
AAEL014137	CLIPB25	CLIPB	CLIPs
AAEL003280	CLIPB26	CLIPB	CLIPs
AAEL007993	CLIPB27	CLIPB	CLIPs
AAEL013245	CLIPB28	CLIPB	CLIPs
AAEL006674	CLIPB29	CLIPB	CLIPs
AAEL000760	CLIPB30	CLIPB	CLIPs
AAEL006161	CLIPB31	CLIPB	CLIPs
AAEL000086	CLIPB32	CLIPB	CLIPs
AAEL000099	CLIPB33	CLIPB	CLIPs
AAEL000028	CLIPB34	CLIPB	CLIPs
AAEL000037	CLIPB35	CLIPB	CLIPs
AAEL005431	CLIPB37	CLIPB	CLIPs
AAEL003628	CLIPB38	CLIPB	CLIPs
AAEL003632	CLIPB39	CLIPB	CLIPs
AAEL003614	CLIPB40	CLIPB	CLIPs
AAEL003631	CLIPB41	CLIPB	CLIPs
AAEL006168	CLIPB42	CLIPB	CLIPs
AAEL014354	CLIPB43	CLIPB	CLIPs
AAEL005060	CLIPB44	CLIPB	CLIPs
AAEL001077	CLIPB45	CLIPB	CLIPs
AAEL005093	CLIPB46	CLIPB	CLIPs
AAEL005064	CLIPB5	CLIPB	CLIPs
AAEL000038	CLIPB6-B36	CLIPB	CLIPs
AAEL003625	CLIPB8	CLIPB	CLIPs
AAEL003610	CLIPB9	CLIPB	CLIPs
AAEL011991	CLIPC1	CLIPC	CLIPs
AAEL011593	CLIPC11	CLIPC	CLIPs
AAEL012711	CLIPC12	CLIPC	CLIPs
AAEL012712	CLIPC13	CLIPC	CLIPs
AAEL004948	CLIPC14	CLIPC	CLIPs
AAEL010270	CLIPC15	CLIPC	CLIPs
AAEL012713	CLIPC16	CLIPC	CLIPs
AAEL007593	CLIPC2	CLIPC	CLIPs
AAEL007597	CLIPC3	CLIPC	CLIPs
AAEL004518	CLIPC5A	CLIPC	CLIPs
AAEL004524	CLIPC5B	CLIPC	CLIPs
AAEL004540	CLIPC6	CLIPC	CLIPs
AAEL007796	CLIPD1	CLIPD	CLIPs
AAEL015109	CLIPD10	CLIPD	CLIPs
AAEL011375	CLIPD11	CLIPD	CLIPs
AAEL004979	CLIPD2	CLIPD	CLIPs

AAEL002997	CLIPD3	CLIPD	CLIPs
AAEL002124	CLIPD6	CLIPD	CLIPs
AAEL015439	CLIPD7	CLIPD	CLIPs
AAEL005906	CLIPD8	CLIPD	CLIPs
AAEL000238	CLIPD9	CLIPD	CLIPs
AAEL010773	CLIPE10	CLIPE	CLIPs
AAEL005800	CLIPE11	CLIPE	CLIPs
AAEL005644	CLIPE12	CLIPE	CLIPs
AAEL005792	CLIPE8	CLIPE	CLIPs
AAEL001233	CLIPE9	CLIPE	CLIPs
AAEL009338	CTL10	CTL	C-Type Lectins
AAEL008299	CTL11	CTL	C-Type Lectins
AAEL008681	CTL12	CTL	C-Type Lectins
AAEL004679	CTL13	CTL	C-Type Lectins
AAEL011453	CTL14	CTL	C-Type Lectins
AAEL012353	CTL15	CTL	C-Type Lectins
AAEL000533	CTL16	CTL	C-Type Lectins
AAEL011446	CTL17	CTL	C-Type Lectins
AAEL005482	CTL18	CTL	C-Type Lectins
AAEL011404	CTL19	CTL	C-Type Lectins
AAEL011407	CTL20	CTL	C-Type Lectins
AAEL011408	CTL21	CTL	C-Type Lectins
AAEL011609	CTL22	CTL	C-Type Lectins
AAEL006456	CTL23	CTL	C-Type Lectins
AAEL002524	CTL24	CTL	C-Type Lectins
AAEL000556	CTL25	CTL	C-Type Lectins
AAEL003119	CTL6	CTL	C-Type Lectins
AAEL010992	CTL8	CTL	C-Type Lectins
AAEL013748	CTL9	CTL	C-Type Lectins
AAEL011402	CTL26	CTL+OTHER	C-Type Lectins
AAEL011078	CTLGA1	CTLGA	C-Type Lectins
AAEL013566	CTLGA2	CTLGA	C-Type Lectins
AAEL011070	CTLGA3	CTLGA	C-Type Lectins
AAEL005641	CTLGA5	CTLGA	C-Type Lectins
AAEL009209	CTLGA6	CTLGA	C-Type Lectins
AAEL011610	CTLGA7	CTLGA	C-Type Lectins
AAEL011619	CTLGA8	CTLGA	C-Type Lectins
AAEL014385	CTLGA9	CTLGA	C-Type Lectins
AAEL011079	CTLMA10	CTLMA	C-Type Lectins
AAEL000543	CTLMA11	CTLMA	C-Type Lectins
AAEL011455	CTLMA12	CTLMA	C-Type Lectins
AAEL011621	CTLMA13	CTLMA	C-Type Lectins
AAEL014382	CTLMA14	CTLMA	C-Type Lectins
AAEL000563	CTLMA15	CTLMA	C-Type Lectins
AAEL000283	CTLMA16	CTLMA	C-Type Lectins
AAEL011612	CTLMA6	CTLMA	C-Type Lectins
AAEL008929	CTLSE1	CTLSE	C-Type Lectins
AAEL014356	CTLSE2	CTLSE	C-Type Lectins
AAEL003841	DEFA	Defensin	AMPs
AAEL003832	DEFC	Defensin	AMPs
AAEL003857	DEFD	Defensin	AMPs

AAEL003849	DEFE	Defensin	AMPs
AAEL001612	DCR1	Dicer	Small regulatory RNA path
AAEL006794	DCR2	Dicer	Small regulatory RNA path
AAEL004833	DPT	Diptericin	AMPs
AAEL012471	DOME	DOME	JAKSTATs
AAEL008592	DROSHA	Drosha	Small regulatory RNA path
AAEL001932	FADD	FADD	IMDPATHs
AAEL009326	FMR1	FMR	Small regulatory RNA path
AAEL010131	FREP1	FREP	Fibrinogen-related proteins
AAEL009723	FREP11	FREP	Fibrinogen-related proteins
AAEL011634	FREP12	FREP	Fibrinogen-related proteins
AAEL011009	FREP13	FREP	Fibrinogen-related proteins
AAEL007942	FREP14	FREP	Fibrinogen-related proteins
AAEL000508	FREP15	FREP	Fibrinogen-related proteins
AAEL011633	FREP16	FREP	Fibrinogen-related proteins
AAEL006704	FREP18	FREP	Fibrinogen-related proteins
AAEL001713	FREP2	FREP	Fibrinogen-related proteins
AAEL000726	FREP20	FREP	Fibrinogen-related proteins
AAEL000749	FREP22	FREP	Fibrinogen-related proteins
AAEL008104	FREP23	FREP	Fibrinogen-related proteins
AAEL013417	FREP24	FREP	Fibrinogen-related proteins
AAEL005194	FREP26	FREP	Fibrinogen-related proteins
AAEL003156	FREP28	FREP	Fibrinogen-related proteins
AAEL013506	FREP29	FREP	Fibrinogen-related proteins
AAEL003294	FREP3	FREP	Fibrinogen-related proteins
AAEL010103	FREP32	FREP	Fibrinogen-related proteins
AAEL006702	FREP33	FREP	Fibrinogen-related proteins
AAEL010117	FREP35	FREP	Fibrinogen-related proteins
AAEL011400	FREP36	FREP	Fibrinogen-related proteins
AAEL011007	FREP37	FREP	Fibrinogen-related proteins
AAEL015428	FREP38	FREP	Fibrinogen-related proteins
AAEL009384	FREP5	FREP	Fibrinogen-related proteins
AAEL004156	FREP9	FREP	Fibrinogen-related proteins
AAEL003840	GALE11	GALE	Galactoside-binding lectins
AAEL009842	GALE12	GALE	Galactoside-binding lectins
AAEL009845	GALE13	GALE	Galactoside-binding lectins
AAEL009850	GALE14	GALE	Galactoside-binding lectins
AAEL012135	GALE2	GALE	Galactoside-binding lectins
AAEL004196	GALE3	GALE	Galactoside-binding lectins
AAEL005294	GALE6A	GALE	Galactoside-binding lectins
AAEL012003	GALE6B	GALE	Galactoside-binding lectins
AAEL005293	GALE8A	GALE	Galactoside-binding lectins
AAEL012001	GALE8B	GALE	Galactoside-binding lectins
AAEL004522	GAM	Gambicin	AMPs
AAEL007626	GNBPA1	GNBPA	GNBPs
AAEL000652	GNBPA2	GNBPA	GNBPs
AAEL003889	GNBPB1	GNBPB	GNBPs
AAEL009176	GNBPB3	GNBPB	GNBPs
AAEL009178	GNBPB4	GNBPB	GNBPs
AAEL003894	GNBPB5	GNBPB	GNBPs
AAEL007064	GNBPB6	GNBPB	GNBPs

AAEL012069	GPXH1	GPX	Peroxidases
AAEL008397	GPXH2	GPX	Peroxidases
AAEL000495	GPXH3	GPX	Peroxidases
AAEL012553	HOP	HOP	JAKSTATs
AAEL003933	DBLOX	HPX	Peroxidases
AAEL007563	DUOX	HPX	Peroxidases
AAEL006014	HPX1	HPX	Peroxidases
AAEL013171	HPX2	HPX	Peroxidases
AAEL005416	HPX3	HPX	Peroxidases
AAEL000376	HPX4	HPX	Peroxidases
AAEL002354	HPX5	HPX	Peroxidases
AAEL012481	HPX6	HPX	Peroxidases
AAEL004401	HPX7	HPX	Peroxidases
AAEL004388	HPX8A	HPX	Peroxidases
AAEL004390	HPX8B	HPX	Peroxidases
AAEL004386	HPX8C	HPX	Peroxidases
AAEL009074	IAP1	IAP	IAPs
AAEL006633	IAP2	IAP	IAPs
AAEL014251	IAP5	IAP	IAPs
AAEL012446	IAP6	IAP	IAPs
AAEL012512	IAP9	IAP	IAPs
AAEL003245	IKK1A	IKKb	IMDPATHs
AAEL010548	IKK1B	IKKb	IMDPATHs
AAEL012510	IKK2	IKKg	IMDPATHs
AAEL010083	IMD	IMD	IMDPATHs
AAEL004392	IMP	IMP	Caspase activators
AAEL008687	LOQS	Loquacious	Small regulatory RNA path
AAEL003712	LYSC10	LYSC	Lysozymes
AAEL003723	LYSC11	LYSC	Lysozymes
AAEL010100	LYSC7A	LYSC	Lysozymes
AAEL015404	LYSC7B	LYSC	Lysozymes
AAEL009670	LYSC9	LYSC	Lysozymes
AAEL005988	LYSC6	LYS-long	Lysozymes
AAEL014196	Michelob_x	Michelob_x	Caspase activators
AAEL004120	ML1	ML	MD2-like receptors
AAEL015135	ML10	ML	MD2-like receptors
AAEL006854	ML13	ML	MD2-like receptors
AAEL009553	ML14A	ML	MD2-like receptors
AAEL015516	ML14B	ML	MD2-like receptors
AAEL009555	ML15A	ML	MD2-like receptors
AAEL009556	ML15B	ML	MD2-like receptors
AAEL015140	ML16	ML	MD2-like receptors
AAEL009557	ML17	ML	MD2-like receptors
AAEL012064	ML2	ML	MD2-like receptors
AAEL015137	ML20	ML	MD2-like receptors
AAEL007592	ML20B	ML	MD2-like receptors
AAEL009760	ML21	ML	MD2-like receptors
AAEL015139	ML22A	ML	MD2-like receptors
AAEL009954	ML22B	ML	MD2-like receptors
AAEL007591	ML26A	ML	MD2-like receptors
AAEL013835	ML26B	ML	MD2-like receptors

AAEL001654	ML30	ML	MD2-like receptors
AAEL001661	ML31	ML	MD2-like receptors
AAEL001634	ML32	ML	MD2-like receptors
AAEL001650	ML33	ML	MD2-like receptors
AAEL015136	ML6	ML	MD2-like receptors
AAEL015138	ML9A	ML	MD2-like receptors
AAEL009953	ML9B	ML	MD2-like receptors
AAEL009531	-	ML	MD2-like receptors
AAEL007768	MYD88	MYD88	TOLLPATHS
AAEL002478	PASHA	Pasha	Small regulatory RNA path
AAEL006571	PELLE	PELLE	TOLLPATHS
AAEL012380	PGRPLA	PGRPL	PGRPs
AAEL010171	PGRPLB	PGRPL	PGRPs
AAEL014640	PGRPLC	PGRPL	PGRPs
AAEL011608	PGRPLD	PGRPL	PGRPs
AAEL013112	PGRPLE	PGRPL	PGRPs
AAEL009474	PGRPS1	PGRPS	PGRPs
AAEL007037	PGRPS4	PGRPS	PGRPs
AAEL007039	PGRPS5	PGRPS	PGRPs
AAEL008076	PIWI1	PIWI	Small regulatory RNA path
AAEL008098	PIWI2	PIWI	Small regulatory RNA path
AAEL013692	PIWI3	PIWI	Small regulatory RNA path
AAEL007698	PIWI4	PIWI	Small regulatory RNA path
AAEL013233	PIWI5	PIWI	Small regulatory RNA path
AAEL013227	PIWI6	PIWI	Small regulatory RNA path
AAEL006287	PIWI7	PIWI	Small regulatory RNA path
AAEL013498	PPO1	PPO	Prophenoloxidases
AAEL011764	PPO10	PPO	Prophenoloxidases
AAEL013499	PPO2	PPO	Prophenoloxidases
AAEL011763	PPO3	PPO	Prophenoloxidases
AAEL013501	PPO4	PPO	Prophenoloxidases
AAEL013492	PPO5	PPO	Prophenoloxidases
AAEL014544	PPO6	PPO	Prophenoloxidases
AAEL013493	PPO7	PPO	Prophenoloxidases
AAEL013496	PPO8	PPO	Prophenoloxidases
AAEL014837	PPO9	PPO	Prophenoloxidases
AAEL011753	R2D2	R2D2	Small regulatory RNA path
AAEL007696	REL1A	REL1	REL
AAEL006930	REL1B	REL1	REL
AAEL007624	REL2	REL2	REL
AAEL001317	RM62A	Rm62	Small regulatory RNA path
AAEL001769	RM62B	Rm62	Small regulatory RNA path
AAEL002083	RM62C	Rm62	Small regulatory RNA path
AAEL002351	RM62D	Rm62	Small regulatory RNA path
AAEL004978	RM62E	Rm62	Small regulatory RNA path
AAEL008738	RM62F	Rm62	Small regulatory RNA path
AAEL010402	RM62G	Rm62	Small regulatory RNA path
AAEL010787	RM62H	Rm62	Small regulatory RNA path
AAEL013985	RM62I	Rm62	Small regulatory RNA path
AAEL001914	SCRAC1	SCRA	Scavenger receptors
AAEL015308	SCRAL1	SCRA	Scavenger receptors

AAEL009192	SCRASP1	SCRA	Scavenger receptors
AAEL010655	SCRASP2	SCRA	Scavenger receptors
AAEL014367	SCRASP3	SCRA	Scavenger receptors
AAEL005374	SCRB1	SCRB	Scavenger receptors
AAEL007748	SCRB10	SCRB	Scavenger receptors
AAEL008370	SCRB17	SCRB	Scavenger receptors
AAEL005987	SCRB2	SCRB	Scavenger receptors
AAEL005979	SCRB3	SCRB	Scavenger receptors
AAEL011222	SCRB5	SCRB	Scavenger receptors
AAEL002741	SCRB6	SCRB	Scavenger receptors
AAEL000234	SCRB7	SCRB	Scavenger receptors
AAEL000227	SCRB8	SCRB	Scavenger receptors
AAEL000256	SCRB9	SCRB	Scavenger receptors
AAEL009420	SCRBQ1	SCRB	Scavenger receptors
AAEL009423	SCRBQ2	SCRB	Scavenger receptors
AAEL009432	SCRBQ3	SCRB	Scavenger receptors
AAEL006355	SCRC1	SCRC	Scavenger receptors
AAEL006361	SCRC2	SCRC	Scavenger receptors
AAEL014091	CuSOD1	SOD-Cu-Zn	Superoxide dismutatses
AAEL006271	CuSOD2	SOD-Cu-Zn	Superoxide dismutatses
AAEL011498	CuSOD3	SOD-Cu-Zn	Superoxide dismutatses
AAEL000259	CuSOD4	SOD-Cu-Zn	Superoxide dismutatses
AAEL004823	MnSOD1	SOD-Mn-Fe	Superoxide dismutatses
AAEL005108	MnSOD2	SOD-Mn-Fe	Superoxide dismutatses
AAEL013235	SPNE	Spindle_E	Small regulatory RNA path
AAEL000499	SPZ1A	SPZ	Spaetzle-like proteins
AAEL013434	SPZ1B	SPZ	Spaetzle-like proteins
AAEL013433	SPZ1C	SPZ	Spaetzle-like proteins
AAEL001435	SPZ2	SPZ	Spaetzle-like proteins
AAEL008596	SPZ3A	SPZ	Spaetzle-like proteins
AAEL014950	SPZ3B	SPZ	Spaetzle-like proteins
AAEL007897	SPZ4	SPZ	Spaetzle-like proteins
AAEL001929	SPZ5	SPZ	Spaetzle-like proteins
AAEL012164	SPZ6	SPZ	Spaetzle-like proteins
AAEL014079	SRPN1	SRPN-INHIB	Serine protease inhibitors
AAEL007765	SRPN10a	SRPN-INHIB	Serine protease inhibitors
AAEL007765	SRPN10b	SRPN-INHIB	Serine protease inhibitors
AAEL007765	SRPN10c	SRPN-INHIB	Serine protease inhibitors
AAEL007765	SRPN10d	SRPN-INHIB	Serine protease inhibitors
AAEL014138	SRPN16	SRPN-INHIB	Serine protease inhibitors
AAEL014078	SRPN2	SRPN-INHIB	Serine protease inhibitors
AAEL002720	SRPN20	SRPN-INHIB	Serine protease inhibitors
AAEL002730	SRPN21	SRPN-INHIB	Serine protease inhibitors
AAEL002715	SRPN22	SRPN-INHIB	Serine protease inhibitors
AAEL005665	SRPN3	SRPN-INHIB	Serine protease inhibitors
AAEL013936	SRPN4a	SRPN-INHIB	Serine protease inhibitors
AAEL013933	SRPN4b	SRPN-INHIB	Serine protease inhibitors
AAEL013937	SRPN4c	SRPN-INHIB	Serine protease inhibitors
AAEL013934	SRPN4d	SRPN-INHIB	Serine protease inhibitors
AAEL014141	SRPN5	SRPN-INHIB	Serine protease inhibitors
AAEL010769	SRPN6	SRPN-INHIB	Serine protease inhibitors

AAEL002699	SRPN7	SRPN-INHIB	Serine protease inhibitors
AAEL011777	SRPN8	SRPN-INHIB	Serine protease inhibitors
AAEL008364	SRPN9	SRPN-INHIB	Serine protease inhibitors
AAEL003686	SRPN11	SRPN-nonINHIB	Serine protease inhibitors
AAEL003653	SRPN12	SRPN-nonINHIB	Serine protease inhibitors
AAEL002731	SRPN14	SRPN-nonINHIB	Serine protease inhibitors
AAEL003697	SRPN17	SRPN-nonINHIB	Serine protease inhibitors
AAEL006137	SRPN19	SRPN-nonINHIB	Serine protease inhibitors
AAEL002704	SRPN23	SRPN-nonINHIB	Serine protease inhibitors
AAEL007420	SRPN25	SRPN-nonINHIB	Serine protease inhibitors
AAEL003182	SRPN26	SRPN-nonINHIB	Serine protease inhibitors
AAEL009692	STAT	STAT	JAKSTATs
AAEL007035	TAK1	TAK1	IMDPATHs
AAEL012267	TEP13	TEP	Thio-ester containing proteins
AAEL014755	TEP15	TEP	Thio-ester containing proteins
AAEL001794	TEP20	TEP	Thio-ester containing proteins
AAEL001802	TEP21	TEP	Thio-ester containing proteins
AAEL000087	TEP22	TEP	Thio-ester containing proteins
AAEL001163	TEP23	TEP	Thio-ester containing proteins
AAEL004000	TOLL10	TOLL	TOLLs
AAEL009551	TOLL11	TOLL	TOLLs
AAEL007613	TOLL1A	TOLL	TOLLs
AAEL003507	TOLL1B	TOLL	TOLLs
AAEL007619	TOLL5A	TOLL	TOLLs
AAEL000057	TOLL5B	TOLL	TOLLs
AAEL000671	TOLL6	TOLL	TOLLs
AAEL002583	TOLL7	TOLL	TOLLs
AAEL000633	TOLL8	TOLL	TOLLs
AAEL013441	TOLL9A	TOLL	TOLLs
AAEL011734	TOLL9B	TOLL	TOLLs
AAEL013528	TPX1	TPX	Peroxidases
AAEL004112	TPX2	TPX	Peroxidases
AAEL014548	TPX3	TPX	Peroxidases
AAEL002309	TPX4	TPX	Peroxidases
AAEL009051	TPX5	TPX	Peroxidases
AAEL011363	TRAF6	TRAF6	TOLLPATHS
AAEL000293	TSN	TSN	Small regulatory RNA path
AAEL007642	TUBE	TUBE	TOLLPATHS
AAEL008073	VIG	VIG	Small regulatory RNA path

TABLE IV

Summary of unmapped and mapped RNA-seq reads. Samstat output summaries and averages of the number of mapped reads under each treatment, as well as the quality statistics for the mapping process. The number of unmapped reads is also noted.

	% from just aligned reads		Nº of reads		% of all reads		
Treatment	% MAPQ>30	MAPQ>30	Unmapped	Total	MAPQ>30	Unmapped	Mapped
Rb24	64.5	11090415	2650685	19833635	56%	13%	87%
Rb36	69.1	10521787	2738074	17969751	59%	15%	85%
Rb48	81.6	12400943	4317882	19512721	64%	22%	78%
Rv24	82.4	12309730	2914234	17856949	69%	16%	84%
Rv36	82.3	10686847	2587216	15577074	69%	17%	83%
Rv48	78.8	9778127	2562484	14969762	65%	17%	83%
Sb24	65.5	11392189	3210246	20596883	55%	16%	84%
Sb36	67.5	6097545	1776032	10807060	56%	16%	84%
Sb48	83.5	11759988	4255288	18331302	64%	23%	77%
Sv24	73	10150845	2543240	16455363	62%	15%	85%
Sv36	79.4	11870160	3085770	18044646	66%	17%	83%
Sv48	80.8	15412758	3257884	22330650	69%	15%	85%

TABLE V

Significantly overrepresented ($p < 0.05$) gene ontology (GO) terms and association number as identified by the Ontologizer (v2.0). Three treatment comparisons are displayed: Cali-S blood fed (Sb) versus Cali-S virus fed (Sv), Cali-R blood fed (Rb) versus Cali-R virus fed (Rv), and Cali-S virus fed (Sv) versus Cali-R virus fed (Rv) at all three time points, 24, 36 and 48 h. Overrepresented upregulated terms appear as green, while downregulated terms appear in red.

A. Molecular function associated GO terms

	Sb vs Sv			Rb vs Rv			Sv vs Rv		
GO term accession and description	4	36	48	24	36	48	24	36	48
GO:0097159|organic cyclic compound binding									
GO:0097367|carbohydrate derivative binding									
GO:1901265|nucleoside phosphate binding									
GO:1901360|organic cyclic metabolic process									
GO:1901363|heterocyclic compound binding									
GO:0000287|magnesium ion binding									
GO:0003723|RNA binding									
GO:0003735|structural constituent of ribosome									
GO:0003779|actin binding									
GO:0003824|catalytic activity									
GO:0005488|binding									

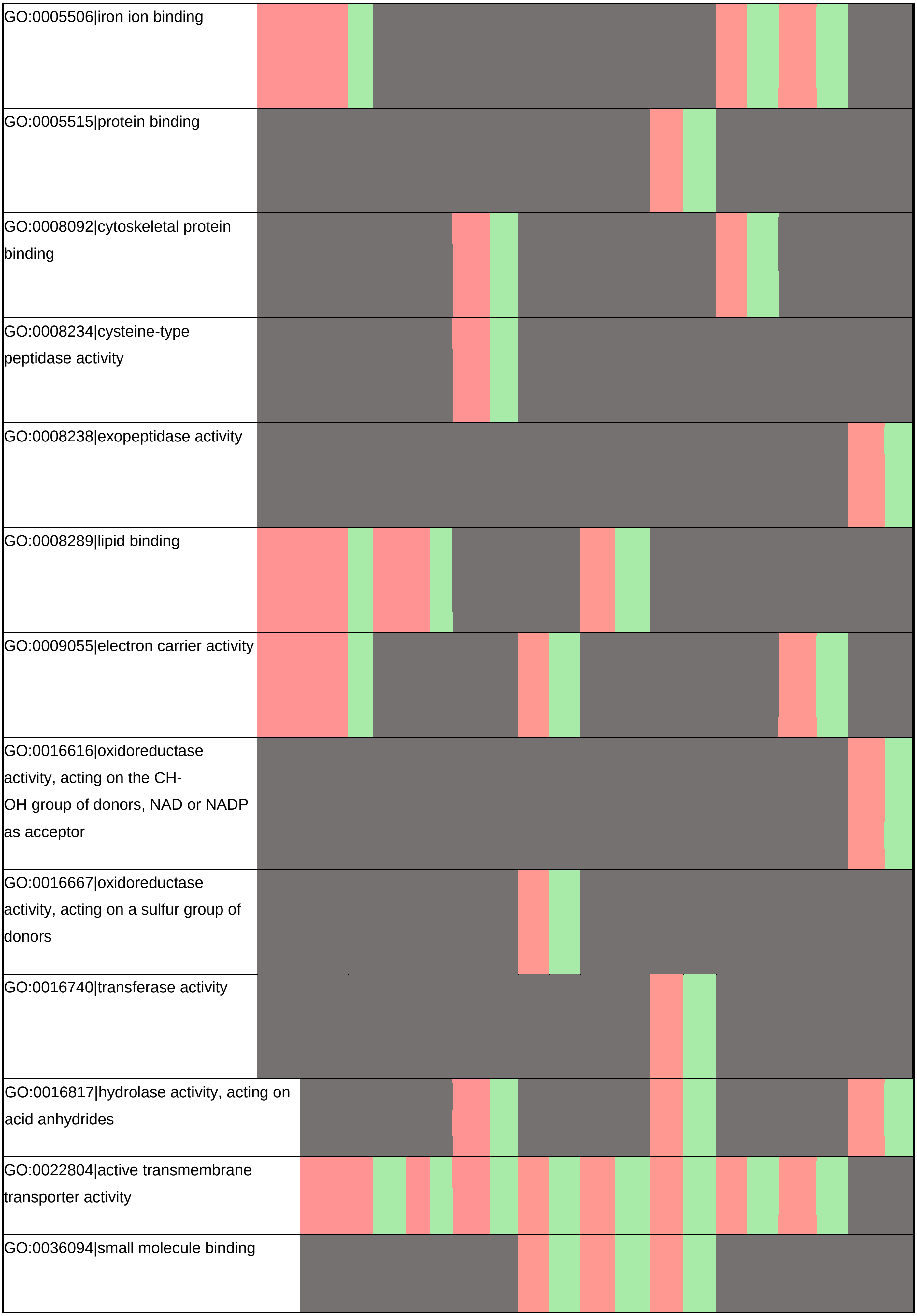
GO:0005506|iron ion binding
GO:0005515|protein binding
GO:0008092|cytoskeletal protein binding
GO:0008234|cysteine-type peptidase activity
GO:0008238|exopeptidase activity
GO:0008289|lipid binding
GO:0009055|electron carrier activity
GO:0016616|oxidoreductase activity, acting on the CH-OH group of donors, NAD or NADP as acceptor
GO:0016667|oxidoreductase activity, acting on a sulfur group of donors
GO:0016740|transferase activity
GO:0016817|hydrolase activity, acting on acid anhydrides
GO:0022804|active transmembrane transporter activity
GO:0036094|small molecule binding

GO:0043167|ion binding	
GO:0043168|anion binding	
GO:0043492|ATPase activity, coupled to movement of substances	
GO:0046906|tetrapyrrole binding	
GO:0048037|cofactor binding	
GO:0051540|metal cluster binding	

B. Biological process associated GO terms

	Sb vs Sv			Rb vs Rv			Sv vs Rv		
GO term accession and description	24	36	48	24	36	48	24	36	48
GO:0065009|regulation of molecular function									
GO:0070887|cellular response to chemical stimulus									
GO:0071702|organic substance transport									
GO:0071826|ribonucleoprotein complex subunit organization									
GO:0071840|cellular component organization or biogenesis									
GO:0098660|inorganic ion transmembrane transport									
GO:1901564|organonitrogen compound metabolic process									
GO:1901700|response to oxygen-containing compound									
GO:0005975|carbohydrate metabolic process									
GO:0006091|generation of precursor metabolites and energy									
GO:0006366|transcription from RNA polymerase II promoter									
GO:0006396|RNA processing									
GO:0006412|translation									
GO:0006793|phosphorus metabolic process									
GO:0006812|cation transport									

GO:0006818|hydrogen transport
GO:0006950|response to stress
GO:0007167|enzyme linked receptor protein signaling pathway
GO:0008150|biological_process
GO:0008152|metabolic process
GO:0009056|catabolic process
GO:0009058|biosynthetic process
GO:0009987|cellular process
GO:0010033|response to organic substance
GO:0010467|gene expression
GO:0010608|posttranscriptional regulation of gene expression
GO:0010646|regulation of cell communication
GO:0016052|carbohydrate catabolic process
GO:0016568|chromatin modification
GO:0019538|protein metabolic process
GO:0022613|ribonucleoprotein complex biogenesis

<table>
<tr><td>GO:0023051|regulation of signaling</td><td></td></tr>
<tr><td>GO:0030203|glycosaminoglycan metabolic process</td><td></td></tr>
<tr><td>GO:0033036|macromolecule localization</td><td></td></tr>
<tr><td>GO:0034660|ncRNA metabolic process</td><td></td></tr>
<tr><td>GO:0035556|intracellular signal transduction</td><td></td></tr>
<tr><td>GO:0043412|macromolecule modification</td><td></td></tr>
<tr><td>GO:0043623|cellular protein complex assembly</td><td></td></tr>
<tr><td>GO:0044281|small molecule metabolic process</td><td></td></tr>
<tr><td>GO:0048583|regulation of response to stimulus</td><td></td></tr>
<tr><td>GO:0051179|localization</td><td></td></tr>
<tr><td>GO:0051641|cellular localization</td><td></td></tr>
<tr><td>GO:0065008|regulation of biological quality</td><td></td></tr>
</table>

C. Cellular component associated GO terms

<table>
<tr><td></td><td colspan="3">Sb vs Sv</td><td colspan="3">Rb vs Rv</td><td colspan="3">Sv vs Rv</td></tr>
<tr><td>GO term accession and description</td><td>24</td><td>36</td><td>48</td><td>24</td><td>36</td><td>48</td><td>24</td><td>36</td><td>48</td></tr>
<tr><td>GO:1990204|oxidoreductase complex</td><td></td><td></td><td></td><td></td><td></td><td></td><td></td><td></td><td></td></tr>
<tr><td>GO:0000502|proteasome complex</td><td></td><td></td><td></td><td></td><td></td><td></td><td></td><td></td><td></td></tr>
<tr><td>GO:0005622|intracellular</td><td></td><td></td><td></td><td></td><td></td><td></td><td></td><td></td><td></td></tr>
</table>

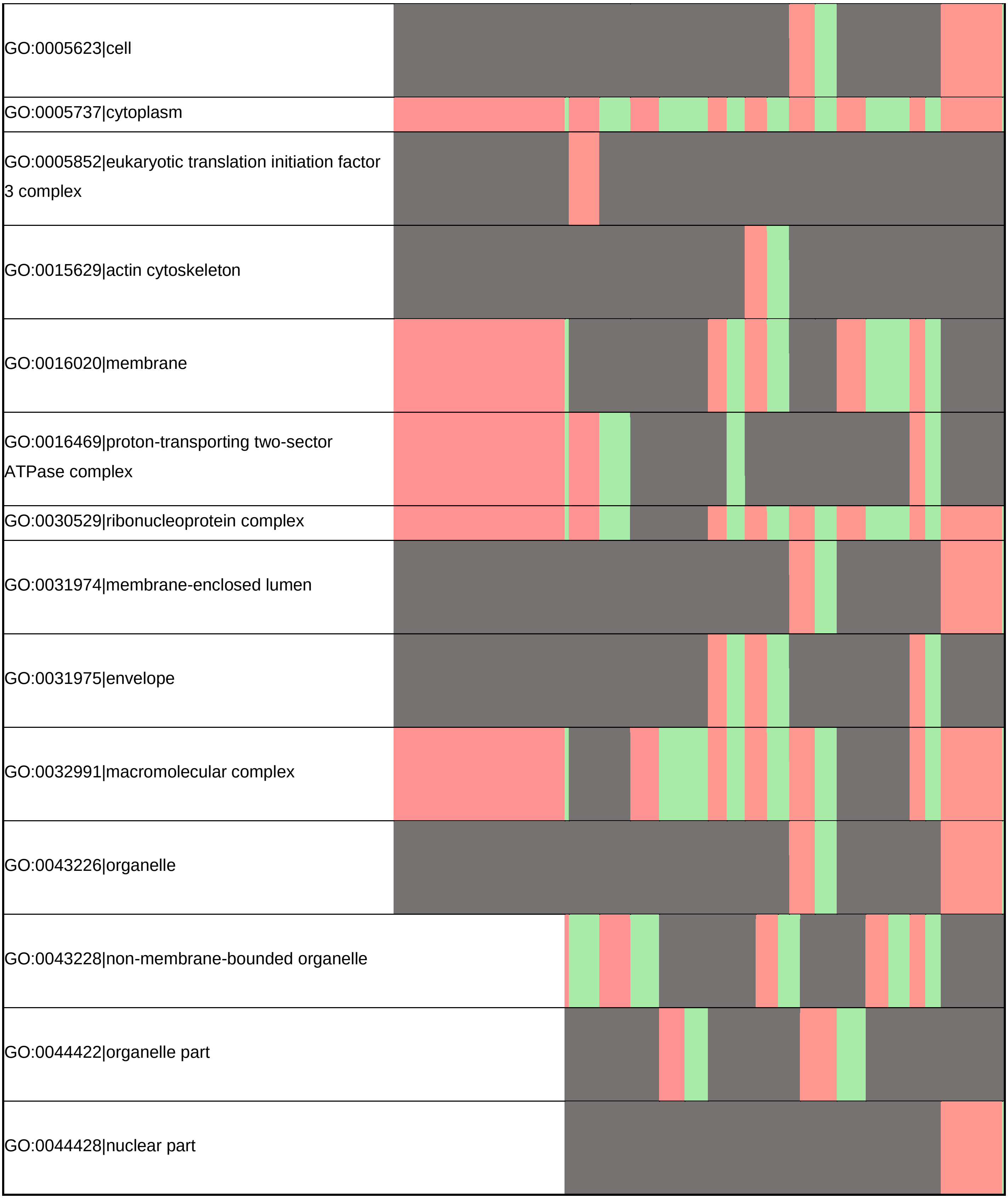
GO:0005623|cell
GO:0005737|cytoplasm
GO:0005852|eukaryotic translation initiation factor 3 complex
GO:0015629|actin cytoskeleton
GO:0016020|membrane
GO:0016469|proton-transporting two-sector ATPase complex
GO:0030529|ribonucleoprotein complex
GO:0031974|membrane-enclosed lumen
GO:0031975|envelope
GO:0032991|macromolecular complex
GO:0043226|organelle
GO:0043228|non-membrane-bounded organelle
GO:0044422|organelle part
GO:0044428|nuclear part
